# Tidal volume and mortality during extracorporeal membrane oxygenation for acute respiratory distress syndrome: a multicenter observational cohort study

**DOI:** 10.1186/s13613-025-01538-9

**Published:** 2025-10-06

**Authors:** Torben M. Rixecker, Jeannine L. Kühnle, Johannes Herrmann, Christopher Lotz, Christian Kühn, Frederik Seiler, Carlos Metz, Armin Kalenka, Oxana Mazuru, Kai Borchardt, Ralf M. Muellenbach, Robert Bals, Matthieu Schmidt, Patrick Meybohm, Vitalie Mazuru, Philipp M. Lepper

**Affiliations:** 1 Department of Internal Medicine V (Pneumology, Allergology and Intensive Care Medicine), Saarland University, Kirrberger Str. 100, 66421 Homburg, Germany; 2 Department of Pneumology and Critical Care Medicine, Cologne-Merheim Hospital, ARDS and ECMO center, University Hospital Würzburg, Kliniken der Stadt Köln, Cologne, Germany; 3 Department of Cardiothoracic, Transplantation and Vascular Surgery, Hannover Medical School, Hannover, Germany; 4 Regional Hospital, Kreiskrankenhaus Bergstrasse, Heppenheim, Germany; 5 Department of Anesthesiology and Critical Care Medicine, ARDS/ECMO-Center, Campus Kassel of the University of Southampton, Kassel, Germany; 6 Helmholtz Institute for Pharmaceutical Research Saarland (HIPS), Helmholtz Centre for Infection Research (HZI), Saarland University Campus, 66123 Saarbrücken, Germany; 7 Sorbonne University, GRC RESPIRE, Medical Intensive Care Unit, Assistance Publique-Hôpitaux de Paris, Pitié-Salpêtrière Hospital, Paris, France; 8 Department of Internal Medicine, Pneumology and Intensive Care Medicine, University Hospital OWL Campus Bethel and University of Bielefeld, Bielefeld, Germany

**Keywords:** ECMO, ARDS, Mortality prediction

## Abstract

**Background:**

Approximately half of the patients with acute respiratory distress syndrome (ARDS) receiving extracorporeal membrane oxygenation (ECMO) remain ECMO-dependent beyond 14 days after ECMO initiation. The identification of factors associated with mortality during an ECMO run may update prognostic assessment and focus clinical interventions.

**Methods:**

In this observational study, data from 1137 patients with COVID-19 ARDS receiving ECMO support in 29 German centers between January 1st 2020 and July 31st 2021 were analyzed. Multivariable stepwise logistic regression analyses were performed to build survival prediction models with day-by-day data during the first 14 days of an ECMO run. The primary endpoint was all-cause mortality in the intensive care unit.

**Results:**

Mortality in this cohort was high (75%). Patients who remained ECMO-dependent on day 14 of their ECMO run showed comparable mortality to all patients receiving ECMO support on day 1. Yet, factors associated with mortality changed during the first 14 days of ECMO support. On day 1 of ECMO support, only patient age and lactate remained in the final mortality prediction model. On day 14 of an ECMO run, tidal volume was independently associated with mortality (adjusted Odds Ratio 0.693 (95%CI 0.564–0.851), p < 0.001 for 1 mL/kg increase in tidal volume per predicted body weight). The adjusted mortality for patients with a tidal volume below 2 mL/kg on day 14 of their ECMO run was above 80% (lower limit of the 95%CI interval). Higher tidal volume was mainly based on higher respiratory system compliance. Yet, the benefit of higher compliance was not observed in some patients who were still ventilated with very low driving pressures despite remaining ECMO-dependent on day 14 of ECMO support.

**Conclusions:**

Mortality predictors change during the course of an ECMO run. In a cohort with high mortality, on day 14 of ECMO support for ARDS, tidal volume may be an independent predictor of mortality. Further analyses on ventilation strategies in patients who remain ECMO-dependent are needed.

**Trial registration number:**

DRKS00022964, retrospectively registered.

**Supplementary Information:**

The online version contains supplementary material available at 10.1186/s13613-025-01538-9.

## Introduction

Patients with severe acute respiratory distress syndrome (ARDS) may benefit from extracorporeal membrane oxygenation (ECMO) [1–4]. Yet, overall mortality for patients receiving ECMO support for ARDS remains high. Therefore, several survival prediction models have been published to improve individual prognostic assessment, but all of them focused on the initiation phase, meaning on the pre-ECMO period [5, 6]. In addition, in the absence of a control group (i.e., patients with similar severity not put on ECMO), the relevance of these predictive survival models has been challenged. Lastly, discriminatory ability is largely moderate and comparable to general intensive care population-based models.

Only a few studies have investigated factors associated with mortality after ECMO initiation [7, 8]. These studies mainly focus on the first few days of ECMO support or evaluate the association between the change in time-dependent covariates throughout an ECMO run and mortality. Still, data on prognostic re-assessment at certain time points during an ECMO run is missing but of high interest, especially when considering that about half of the patients remain ECMO-dependent beyond 14 days after ECMO initiation as reported in the EOLIA trial and other observational ECMO studies [9, 10].

Significant uncertainty surrounds the use of ECMO as a bridge to lung transplantation for patients with refractory ARDS [11]. Careful patient selection is essential to ensure potential benefits. Clinically, the appropriate duration of ECMO remains a subject of ongoing debate, particularly regarding its potential to facilitate successful lung recovery. So far, the decision to transition from a bridge-to-recovery strategy to a lung transplant approach is not based on a few isolated factors.

Thus, the aim of this study was to identify predictive factors for mortality at certain time points during an ECMO run, simulating a clinical round of an ECMO patient, in order to update prognostic assessment, contribute to the decision on continuing therapy and focus clinical interventions.

## Methods

### Cohort

Independent survival prediction models were derived from 1137 patients with COVID-19 ARDS receiving ECMO support between January 1 st 2020, and July 31 st, 2021 in 29 ECMO centers across Germany included in the German COVID-19 ECMO registry. This registry continuously collects observational, multi-center data to recognize structural- and patient-related risk factors, complications, treatment effects, and their outcome [12]. The treating physicians documented data in the register using a standardized electronic case report form (RedCap, Vanderbilt University, Nashville, TN). Patients with missing data on the primary outcome, patients who were transferred to another ICU and patients with arterial cannulation were excluded from our analyses.

### Independent variables and outcome

The primary outcome was all-cause mortality in the intensive care unit (ICU mortality) at any time.

The independent variables tested as predictors included: ECMO blood flow, ECMO sweep gas flow, PaO_2_, PaCO_2_, lactate, pH, renal replacement therapy (RRT), age, time between intubation and ECMO onset and Sequential Organ Failure Assessment (SOFA) Score.

In patients under controlled ventilation, the following ventilation variables were included: respiratory rate, inspiratory pressure (P_high_), positive end-expiratory pressure (PEEP), driving pressure (P_high_ minus PEEP), tidal volume per predicted body weight (Vt/PBW), respiratory system compliance (C_rs_) calculated as Vt/driving pressure. PEEP was also recorded in patients receiving spontaneous ventilation.

Ventilation modes were classified as spontaneous, assisted or controlled. However, information on whether pressure-targeted or volume-targeted modes were used was not available. All of these covariates were determined on a day-by-day basis and recorded at a single time point per observational day.

Independent survival prediction models for days 1, 3, 5, 7 and 14 of an ECMO run with these covariates were built based on the data of all patients who were still receiving ECMO support on the respective days. Patients who died or were liberated from the ECMO before the respective day of their ECMO run were not considered for the respective model of this day (censored). Thus, our study simulates the clinical round of an ECMO patient. Focusing on survival prediction for patients who remain ECMO-dependent, models for day 1 and day 14 were primarily considered.

### Statistical analysis

We followed the STROBE (Strengthening the Reporting of Observational Studies in Epidemiology) recommendations for reporting cohort studies [13].

Variables were expressed as median (25th–75th percentiles). Comparisons between continuous variables were performed using the paired Wilcoxon signed-rank test, and comparisons between independent groups were made using the Wilcoxon rank-sum tests. Categorical variables were compared with chi-square tests or Fisher exact tests.

Survival prediction models were built independently for all ECMO days using multivariable stepwise logistic regression analysis. For this purpose, all clinically relevant factors (achieving p ≤ 0.20) in univariable analysis were subsequently entered into a forward stepwise multivariate analysis and then into a backward stepwise multivariate analysis. Variables that were consistently found to be associated with survival (p < 0.05) with the use of both modeling procedures were included in the final models. Multicollinearity was assessed by calculating a variance inflation factor of each variable and ruled out if the variance inflation factor was lower than 4. The final models were based on data from patients with complete information available for all variables. These models were re-run after multiple imputation for missing data (pooled results from 20 imputed datasets are shown).

To illustrate the prognostic value of tidal volume on day 14, we conducted three separate K-means cluster analyses (k = 2) on the data set of all patients still receiving ECMO support based on driving pressure, tidal volume, and respiratory system compliance (C_rs_), respectively, and compared the adjusted odds ratios for ICU mortality across the resulting clusters.

The course of daily collected data over time was analyzed using repeated measures ANOVA analysis.

Results are reported with 95% confidence interval. Statistical analyses were performed with SPSS 29.0 (IBM Statistics, Amok, USA). All P values were two-sided, with p < 0.05 considered statistically significant.

### Ethics

The study protocol for the German COVID-19 ECMO registry was approved by the Ethics Committee of the Medical Faculty of the Julius-Maximilians-University of Würzburg (131/20-me). Additional local ethics committee votes were obtained from each of the participating ECMO centers. The register was registered in the German Clinical Trials Register (study ID: DRKS00022964, retrospectively registered, September 7th 2020).

According to German legislation, no informed consent for retrospective, anonymous data is required and informed consent was waived by the ethics committee.

## Results

### Patient characteristics

**Fig. 1:**
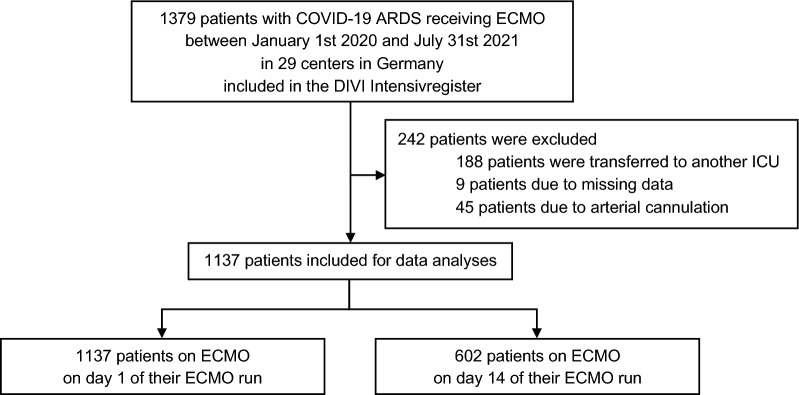
Study Flow Chart. Definition of abbreviations: ECMO = extracorporeal membrane oxygenation; ARDS = acute respiratory distress syndrome

A study flow diagram is presented in Fig. 1. 53% of all patients remained under ECMO support 14 days after ECMO initiation. ICU mortality for patients still receiving ECMO support on day 14 was comparable to the mortality of all patients receiving ECMO support on day 1: 75% and 72% for days 1 and 14, respectively.

Patient characteristics for all patients on ECMO on day 1 and for those still receiving ECMO support on day 14 according to survival status are summarized in Table 1.

**Table 1:** Characteristics of all Patients on ECMO

	Day 1 of ECMO*			Day 14 of ECMO^#^		
	Survivors	Non-Survivors	*p*-value	Survivors	Non-Survivors	*p*-value
	N = 282	N = 855		N = 167	N = 435	
Age/Age Group	< 41 (17%) 41–70 (81%) > 70 (2%)	< 41 (8%) 41–70 (86%) > 70 (6%)	< 0.001	< 41 (16%) 41–70 (83%) > 70 (1%)	< 41 (10%) 41–70 (86%) > 70 (4%)	< 0.001
ECMO blood Flow (lpm)	4.0 (3.5;4.5)	4.1 (3.5;4.6)	0.163	3.8 (3.1;4.5)	4.5 (3.8;5.3)	< 0.001
ECMO sweep Flow (lpm)	4 (3;5)	4 (3;5)	0.784	5 (3;7)	6 (5;8)	< 0.001
Ventilation mode Spontaneous Assisted Controlled	20 (7%) 44 (16%) 218 (77%)	19 (2%) 99 (12%) 737 (86%)	< 0.001	18 (11%) 37 (22%) 112 (67%)	20 (5%) 81 (19%) 333 (76%)	0.009
Respiratory rate (breaths/minute)	16 (12;20)	15 (12;20)	0.012	20 (15;25)	18 (13;25)	0.054
P_high_ (cmH_2_O)	27 (24;30)	27 (24;30)	0.640	26 (24;30)	28 (25;30)	0.037
PEEP (cmH_2_O)	14 (12;15)	14 (12;16)	0.406	12 (10;14.3)	14 (11;16)	< 0.001
Driving pressure (cmH_2_O)	12 (10;15)	13 (10;15)	0.654	14 (12;17)	14 (11;17)	0.254
Tidal volume (ml/kg)	3.7 (2.5;5.2)	3.7 (2.7;5.0)	0.825	3.5 (2.3;5.2)	2.6 (1.4;4.2)	< 0.001
Compliance (ml/cmH_2_O)	21.3 (13;32)	21.7 (14;30)	0.906	17.7 (11;25)	13.8 (8;22)	0.009
PaO_2_ (mmHg)	76 (68;95)	76 (67;76)	0.164	76 (65;88)	71.75 (63;83)	0.004
PaCO_2_ (mmHg)	47 (42;52)	47.4 (42;54)	0.125	46.8 (43;53)	47 (42;53)	0.991
Arterial pH	7.4 (7.35;7.40)	7.39 (7.33;7.40)	0.318	7.39 (7.35;7.40)	7.38 (7.34;7.40)	0.006
Lactate (mmol/L)	1.5 (1.2;2.1)	1.8 (1.3;2.5)	< 0.001	1.0 (0.7;1.4)	1.3 (1;2)	< 0.001
SOFA	11 (8;13)	12 (9;15)	0.054	11 (8;13)	13 (10;16)	< 0.001

ECMO = extracorporeal membrane oxygenation; P_high_ = inspiratory pressure; PEEP = positive end expiratory pressure; Vt = tidal volume; PBW = predicted body weight; PaO_2_ = arterial partial pressure of oxygen; PaCO_2_ = arterial partial pressure of carbon dioxide, SOFA = sequential organ failure assessment

Results are expressed as median (IQR), mean (± SD) or %

*Data available for 1137 (Age Group), 1119 (ECMO Blood Flow, ECMO Sweep Flow), 913 (Respiratory Rate), 927 (P_high_, PEEP, Driving Pressure), 841 (Vt/PBW, Compliance), 1138 (PaO_2_, PaCO_2_, Arterial pH, Lactate), 842 (SOFA) patients

#Data available for 602 (Age, ECMO Flow, ECMO Sweep), 422 (Respiratory Rate), 405 (P_high_, PEEP, Driving Pressure, VT/PBW, Compliance), 601 (PaO2, PaCO2, arterial pH, Lactate) and 439 (SOFA) patients

Non-Survivors on both days of ECMO support were older, had higher lactate concentrations and received controlled ventilation more frequently.

On both days of ECMO support, median inspiratory pressures above 25cmH_2_O were observed.

Respiratory system compliance was calculated for patients receiving controlled ventilation only and more than two thirds of all patients were still under controlled ventilation on day 14 of their ECMO run.

While compliance and tidal volume on day 1 of an ECMO run did not differ between survivors and non-survivors, on day 14 of an ECMO run, survivors showed a higher compliance and higher tidal volumes.

In patients, who remained ECMO-dependent, tidal volumes declined, although increasing driving pressures were applied (Supplementary Figure S1), supporting the general use of pressure-controlled ventilation.

### Prediction models

In univariable analyses several candidate variables qualified for multivariable assessment (Supplementary Tables S1). Complete data sets for days 1 and 14 of an ECMO run were available for 56% and 42% of all patients, respectively. Variables significantly associated with ICU mortality after multivariable adjustment and sensitivity analyses for missing data using multiple imputations on day 1 and day 14 are shown in Table 2 (independent Models for all days are shown in Supplementary Table S2).

**Table 2:** Multivariable ICU Mortality Prediction Models for all Patients on ECMO

	Stepwise regression models (listwise deletion)			
	Day 1 of ECMO		Day 14 of ECMO	
	n = 642		n = 250	
	Odds Ratio (95% CI)	*p*-value	Odds Ratio (95% CI)	*p*-value
Age group	1.507 (1.332–1.705)	< 0.001		
Lactate on this day (mM)	1.164 (1.072–1.265)	< 0.001	2.442 (1.569–3.801)	< 0.001
ECMO blood Flow on this day (Lpm)			1.247 (1.004–1.548)	0.046
Tidal volume on this day (mL/kg)			0.693 (0.564–0.851)	< 0.001

	Sensitivity analysis (multiple imputation*)			
	Day 1 of ECMO		Day 14 of ECMO	
	n = 1137		n = 602	
	Odds Ratio (95% CI)	*p*-value	Odds Ratio (95% CI)	*p*-value
Age group	1.515 (1.321–1.738)	< 0.001		
Lactate on this day (mM)	1.164 (1.061–1.275)	0.001	1.747 (1.294–2.459)	< 0.001
ECMO blood Flow on this day (Lpm)			1.390 (1.171–1.650)	< 0.001
Tidal volume on this day (mL/kg)			0.882 (0.796–0.978)	0.017

*pooled results from 20 imputed datasets are shown

ECMO = extracorporeal membrane oxygenation

On day 1, only patient age and lactate were independently associated with mortality. In the following days, ECMO blood flow emerged as an independent mortality predictor with higher ECMO blood flows being associated with higher mortality.

**Fig. 2:**
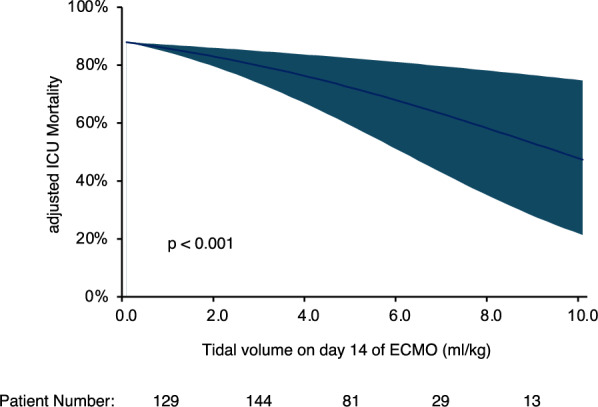
Adjusted ICU Mortality based on Tidal Volume on Day 14 of an ECMO run for Patients still receiving ECMO support. Adjusted ICU Mortality based on tidal volume per predicted body weight on day 14 of an ECMO run for patients under controlled ventilation still receiving ECMO support. ICU Mortality was adjusted for all relevant covariates based on stepwise regression analysis as shown in Table 2 (ECMO blood flow on day 14 and lactate on day 14)

On day 14 of an ECMO run, tidal volume was the first ventilatory parameter to emerge as a prognostic factor in these analyses. The adjusted mortality for all patients still receiving ECMO support on day 14 based on tidal volume is shown in Fig. 2. Patients with a tidal volume below 2 mL/kg predicted body weight on this day of their ECMO run showed an adjusted mortality above 80% (lower limit of the 95% confidence interval).

### Independence of information

Although tidal volume and compliance are mathematically linked, and a lower mortality in patients with higher tidal volume could be the result of improved compliance, collinearity between these predictor variables was moderate (VIF = 1.653), and compliance was not associated with mortality when forced into the day 14 model with tidal volume already being a covariate (Table 3).

**Table 3:** Tidal Volume and Compliance on Day 14 of ECMO support

Day 14 Model 2 (Forcing C_rs_ into the model)		
n = 250		
	Odds Ratio (95% CI)	*p*-value
Lactate on this day (mM)	2.415 (1.560–3.740)	< 0.001
ECMO blood flow on this day (Lpm)	1.241 (0.999–1.542)	0.051
Tidal volume on this day (mL/kg)*	0.784 (0.668–0.919)	0.003
Compliance on this day (mL/cmH_2_O)*	1.008 (0.986–1.031)	0.473

Day 14 Model 2 sensitivity analysis (Forcing C_rs_ into the model)		
n = 602		
	Odds Ratio (95% CI)	*p*-value
Lactate on this day (mM)	1.743 (1.290–2.354)	< 0.001
ECMO blood flow on this day (Lpm)	1.385 (1.165–1.646)	< 0.001
Tidal volume on this day (mL/kg)^#^	0.869 (0.762–0.992)	0.038
Compliance on this day (mL/cmH_2_O)^#^	1.003 (0.985–1.021)	0.747

Vt = tidal volume; PBW = predicted body weight; C_rs_ = respiratory system compliance

*Vt/PBW and C_rs_ had a variance inflation factor (VIF) of 1.653

#Vt/PBW and C_rs_ had a variance inflation factor (VIF) < 2 in all 20 imputed data sets

In patients with additional spontaneous breathing however, calculated compliance may not correspond to actual compliance, with the actual compliance being presumably overestimated by calculation from driving pressure and tidal volume. To address this issue, in the abscence of data on more specific markers for spontaneous breathing (e.g. difference between set and actual respiratory rate, transpulmonary pressure or airway occlusion pressure (P0.1)) we forced respiratory rate as continuous and dummy variable with various thresholds into the day 14 model (Supplementary Table S3). In this analysis, a respiratory rate ≥ 14/min improved the model fit and was associated with a non-significantly lower mortality itself. Still, the association between tidal volume and mortality remained stable regardless of how respiratory rate was modeled. In addition, tidal volume retained prognostic priority over respiratory system compliance when forcing both compliance and respiratory rate ≥ 14/min into the day 14 model (Supplementary Table 4). This result also remained unchanged when including the ECMO center as a covariate, acknowledging that ventilation strategies may differ between centers (data not shown).

### Tidal volume, compliance and driving pressure on day 14 of ECMO support

**Fig. 3:**
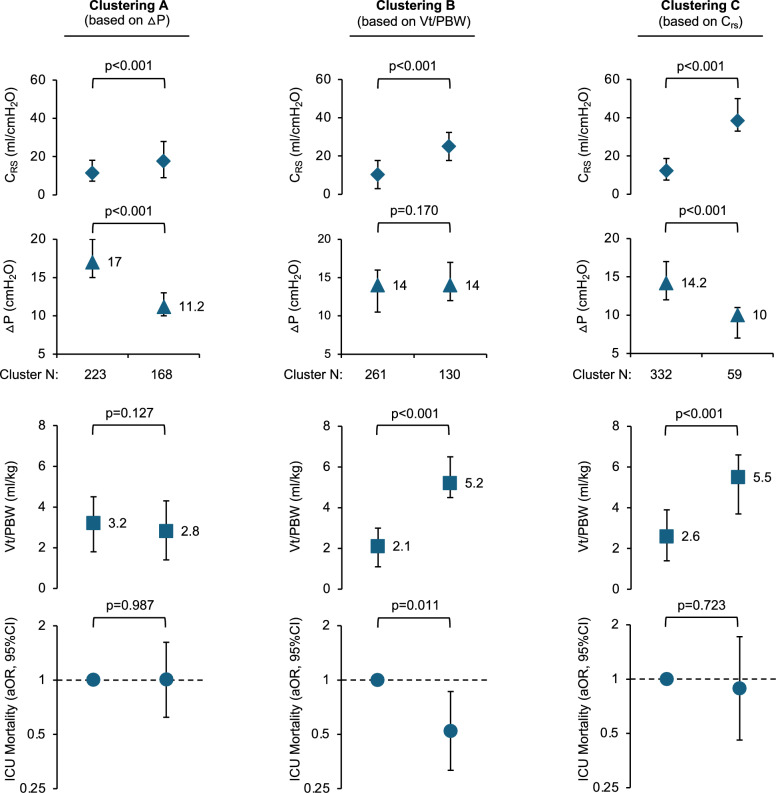
Tidal Volume, Compliance and Driving Pressure on Day 14 of ECMO support. Using K-means cluster analysis (k = 2) we partitioned our data set of all patients under controlled ventilation still receiving ECMO support on day 14 of their ECMO run into clusters based on driving pressure (ΔP) (Clustering A), tidal volume (Vt/PBW) (Clustering B) and respiratory system compliance (Clustering C) and calculated the adjusted odds ratio for mortality. The upper scatter/error-bar diagrams illustrate the median values observed in each cluster. The error bars represent interquartil ranges. At the bottom, the respective adjusted odds ratios for ICU mortality are shown, calculated for each cluster after multivariable adjustment for all relevant covariates based on stepwise regression analysis as shown in Table 2 (ECMO blood flow on day 14 and lactate on day 14). Error bars represent 95% confidence intervals. Definition of abbreviations: Vt = tidal volume; PBW = predicted body weight; C_rs_ = respiratory system compliance; ΔP = driving pressure; aOR = adjusted odds ratio

To compare the impact of tidal volume, respiratory system compliance and driving pressure on day 14 of an ECMO run, we resampled the datasets of all patients still receiving ECMO support with data on the final covariates (ECMO blood flow on this day, lactate on this day, tidal volume on this day, compliance on this day), forming clusters of patients based on the variables driving pressure (Clustering A), tidal volume (Clustering B) and respiratory system compliance (Clustering C), respectively (Fig. 3).

In this analysis, an effect on the adjusted ICU mortality was observed only when clustering was performed based on tidal volume (Clustering B). In this clustering, higher tidal volume was mainly based on higher respiratory system compliance with comparable levels of driving pressure between clusters. However, the benefit of higher respiratory system compliance was not observed in some patients (n = 59), who were ventilated with very low driving pressures (10 (7–11) cmH_2_O versus 14.2 (12–17) cmH_2_O for median (IQR)), diminishing the effect on tidal volume (Clustering C).

## Discussion

In this study, conducted on a large multicenter cohort of patients with severe COVID-19 related ARDS receiving ECMO support, we found that predictors of mortality change during an ECMO run. While, consistent with previous reports [7], ventilatory parameters at the beginning of an ECMO run were comparable between survivors and non-survivors, on day 14 of ECMO support, tidal volume was identified as an independent predictor of mortality, with decreasing tidal volume being increasingly linked to higher mortality.

Higher tidal volume was mainly based on higher respiratory system compliance. Why some patients with high respiratory system compliance, who remained ECMO-dependent, were still ventilated with very low driving pressures remains unknown. The widespread assumption that driving pressure is invariably harmful might have contributed to this. Yet, both potential confounders and miscalculations of the actual compliance based on the available data must be acknowledged, which precludes drawing firm conclusions. Still, this analysis reflects the real-world for clinicians, in which respiratory system compliance, in contrast to tidal volume, will always be a calculated measure.

While the importance of lowering driving pressure in the early phase of an ECMO run and ARDS is well documented [14, 15], data on ventilation settings in patients who remain ECMO dependent is missing. Our study may suggest that there is a point in time in patients with ARDS who remain ECMO-dependent, when achieving higher tidal volumes might be preferable to the continuation of ultra-protective lung ventilation with very low driving pressures [16], especially when a high risk of mortality is anticipated.

Usually, no later than after 4 weeks of ECMO support, concerns emerge regarding the lung's potential for recovery, leading to lung transplantation being considered as the only viable option for survival. However, it even remains unclear what constitutes prolonged ECMO support, with no clear consensus on how long such support can be beneficial [17]. Additionally, significant concerns persist regarding the criteria for defining irreversible lung impairment, even when assessed by a multidisciplinary team in patients with severe ARDS lasting more than 4 weeks. Consequently, our study provides valuable insights into what outcomes may be anticipated after 14 days of ECMO support. This is especially critical, given the high stakes involved. Key clinical decisions, such as whether to discontinue therapy or pursue maximal interventions like lung transplantation, must be considered. However, organ availability is limited, and lung transplantation in patients with ARDS on ECMO is both rare and associated with significant risks. While the decision will not be based solely on a prediction model, the data presented here suggest to consider lung recruitment starting from day 14 of ECMO support.

Using day-by-day data from a large cohort of patients with ARDS on ECMO, we investigated survival prediction models at certain time points during the first 14 days of an ECMO run simulating a medical round of ECMO patients. This approach differs from previous studies using cox regression models with time-dependent covariates, showing that improvements in tidal volume [7] or compliance [18] during an ECMO run are associated with better survival. First, although time-dependent covariates in cox regression represent a very powerful statistical method for modeling and capturing the impact of time-variying factors, their interpretation remains challenging in routine clinical practice. Second, modeling tidal volume or compliance with a linear time function may fail to capture more complex, non-linear dynamics, especially when comparing these covariates. Specifically, as disease physiology changes [19], and the ECMO run continues, the risk-to-benefit profile of continuing ultra-protective lung ventilation [16] and the relative prognostic value when comparing compliance with tidal volume may shift. Simulating a medical round of ECMO patients may offer an initial, simply structured framework for this comparison.

Our study has several limitations. First, although the comparison of tidal volume and respiratory system compliance only involved patients under controlled ventilation, we had no data on pressures across the lung (transpulmonary ΔP), additional spontaneous breathing efforts, airway occlusion pressure (P0.1) or sedation, factors potentially associated with tidal volume, compliance and mortality, thus serving as potential confounders. These results should therefore be interpreted with caution as recovery might be overestimated in the group of patients with high calculated respiratory system compliance (clustering C), potentially explaining the lack of effect on mortality. The persistent priority of tidal volume when compared to respiratory system compliance despite the additional inclusion of respiratory rate ≥ 14 as a surrogate for spontaneous breathing into the day 14 model can only partially address this limitation.

Second, we had no data on mode of ventilation (pressure-targeted vs. volume-targeted) and can only assume that most of these patients on ECMO were ventilated in a pressure-targeted mode as previously described, which would imply that inspiratory pressure (P_high_) may closely reflect plateau pressure and that the calculated compliance corresponds to static compliance [7]. This assumption is supported by the individually varying tidal volumes over time despite increasing driving pressures (Supplementary Figure S1), which would not be expected under volume-targeted ventilation, even with pressure limitation.

Third, only 56% and 42% of all patients on ECMO had complete data sets considering days 1 and 14 of an ECMO run, respectively, limiting the validity of our results. Fourth, most variables were collected as point data once a day, which may not accurately reflect values considering the entire day. Last, ICU mortality in this cohort of patients with COVID-19 ARDS early in the course of the pandemic was high as discussed previously [12], especially when compared to other analyses focusing on mortality predictors during an ECMO run [7, 15, 18]. Thus, the findings presented here, in particular those concerning the comparison of tidal volume and compliance may not apply to patients with better survival or non-COVID-19 ARDS under ECMO support.

Nevertheless, our results provide new insights that could help assess the prognosis of patients who remain dependent on ECMO support by day 14 of their ECMO run. They offer valuable guidance on key factors to focus on, contributing to a more informed approach to patient management in this critical phase.

## Conclusions

Mortality predictors change during the course of an ECMO run. In patients with ARDS, who remain ECMO-dependent beyond 14 days after ECMO initiation, tidal volume may be an independent predictor of mortality.

## Supplementary Information

Additional file 1.

## Data Availability

The data that support the findings of this study are available from the corresponding author, upon reasonable request.
