# Supplementary material for: Tidal volume and mortality during extracorporeal membrane oxygenation for acute respiratory distress syndrome: a multicenter observational cohort study
Source: Ann Intensive Care. 2025 Oct 6;15:151. doi: 10.1186/s13613-025-01538-9 (PMC12500509; doi:10.1186/s13613-025-01538-9)
Supplement: Supplementary file 1 — Additional file 1. [file 13613_2025_1538_MOESM1_ESM.docx]

**Supplementary Information**

**Tidal Volume and Mortality during Extracorporeal Membrane Oxygenation for Acute Respiratory Distress Syndrome: A multicenter observational cohort study**

Torben M. Rixecker^1^*, Jeannine L. Kühnle^1^*, Johannes Herrmann^2^, Christopher Lotz^2^, Christian Kühn^3^, Frederik Seiler^1^, Carlos Metz^1^, Armin Kalenka^4^, Oxana Mazuru^1^, Kai Borchardt^1^, Ralf M. Muellenbach^5^, Robert Bals^1,6^, Matthieu Schmidt^7^, Patrick Meybohm^2^, Vitalie Mazuru^1^* and Philipp M. Lepper^1,8^*

for the German ECMO COVID Study Group

*^1^ Department of Internal Medicine V (Pneumology, Allergology and Intensive Care Medicine), University Medical Center and Saarland University, Germany*

*^2^ University Hospital Würzburg, Department of Anaesthesiology, Intensive Care, Emergency and Pain Medicine, Würzburg, Germany*

*^3^ Department of Cardiothoracic, Transplantation and Vascular Surgery, Hannover Medical School, Hannover, Germany*

*^4^ Regional Hospital, Kreiskrankenhaus Bergstrasse, Heppenheim*

*^5^* *Department of Anesthesiology and Critical Care Medicine, ARDS/ECMO-Center, Campus Kassel of the University of Southampton, Kassel, Germany.*

*^6^ Helmholtz Institute for Pharmaceutical Research Saarland (HIPS), Helmholtz Centre for Infection Research (HZI), Saarland University Campus, 66123 Saarbrücken, Germany.*

*^7^* *Sorbonne University, GRC RESPIRE, Medical Intensive Care Unit, Assistance Publique-Hôpitaux de Paris, Pitié-Salpêtrière Hospital, Paris, France*

*^8^Department of Internal Medicine, Pneumology and Intensive Care Medicine, University Hospital OWL Campus Bethel and University of Bielefeld, Germany*

** these authors contributed equally*

**Table of contents**

| **Title** | **Page** |
| --- | --- |
|  |  |
| Table S1: Univariable Binary Logistic Regression Models for all Days | 3-5 |
| Table S2: Multivariable Stepwise Regression Models for all Days | 6 |
| Table S3. Multivariable Model including Respiratory Rate for all Patients still receiving ECMO support on day 14 | 7-8 |
| Table S4. Multivariable Model including Respiratory Rate (≥ 14/min) and Respiratory System Compliance for all Patients still receiving ECMO support on day 14 | 9 |
| Figure S1. Tidal Volumes and Driving Pressures during the ECMO runs | 10 |

**Table S1.** Binary logistic regression analyses

**Day 1**

| **Variable** | **Odds Ratio** | **p-value** |
| --- | --- | --- |
| Age | 1.509 | < 0.001 |
| BMI | 0.984 | 0.049 |
| Immunodeficiency | 1.006 | 0.032 |
| SOFA d1 | 1.040 | 0.047 |
| Days Intubation to ECMO | 1.060 | <0.001 |
| Tidal volume d1 | 1.000 | 0.606 |
| Respiratory frequency d1 | 0.973 | 0.022 |
| Driving pressure d1 | 1.007 | 0.676 |
| Inspiratory Pressure (P_high_) d1 | 1.008 | 0.642 |
| PEEP d1 | 1.001 | 0.957 |
| Compliance d1 | 0.999 | 0.752 |
| Dialysis d1 | 1.860 | < 0.001 |
| pH d1 | 0.366 | 0.217 |
| Lactate d1 | 1.173 | < 0.001 |
| ECMO Flow d1 | 1.100 | 0.217 |
| Sweep Gas Flow d1 | 1.005 | 0.888 |
| Tidal volume per predicted body weight d1 | 0.964 | 0.590 |

**Day 3**

| **Variable** | **Odds Ratio** | **p-value** |
| --- | --- | --- |
| Age | 1.509 | < 0.001 |
| BMI | 0.984 | 0.049 |
| Immunodeficiency | 1.006 | 0.032 |
| SOFA d3 | 1.055 | 0.007 |
| Days Intubation to ECMO | 1.060 | <0.001 |
| Vt/PBW d3 | 0.999 | 0.033 |
| Respiratory frequency d3 | 0.998 | 0.843 |
| Driving pressure d3 | 1.009 | 0.585 |
| Inspiratory Pressure (P_high_) d3 | 1.037 | 0.057 |
| PEEP d3 | 1.027 | 0.168 |
| Compliance d3 | 0.997 | 0.447 |
| Dialysis d3 | 1.535 | 0.008 |
| pH d3 | 0.127 | 0.050 |
| Lactate d3 | 1.207 | 0.005 |
| ECMO Flow d3 | 1.343 | < 0.001 |
| Sweep Gas Flow d3 | 1.138 | < 0.001 |
| Tidal volume per predicted body weight d3 | 1.010 | 0.907 |

**Day 5**

| **Variable** | **Odds Ratio** | **p-value** |
| --- | --- | --- |
| Age | 1.509 | < 0.001 |
| BMI | 0.984 | 0.049 |
| Immunodeficiency | 1.006 | 0.032 |
| SOFA d5 | 1.069 | 0.001 |
| Days Intubation to ECMO | 1.060 | <0.001 |
| Vt/PBW D5 | 0.998 | <0.001 |
| Respiratory frequency d5 | 1.005 | 0.671 |
| Driving pressure d5 | 1.029 | 0.080 |
| Inspiratory Pressure (P_high_) d1d5 | 1.053 | 0.005 |
| PEEP d5 | 1.009 | 0.645 |
| Compliance d5 | 0.991 | 0.040 |
| Dialysis d5 | 1.451 | 0.022 |
| pH d5 | 0.020 | <0.001 |
| Lactate d5 | 1.186 | 0.014 |
| ECMO Flow d5 | 1.482 | <0.001 |
| Sweep Gas Flow d5 | 1.175 | <0.001 |
| Tidal volume per predicted body weight d5 | 0.809 | 0.003 |

**Day 7**

| **Variable** | **Odds Ratio** | **p-value** |
| --- | --- | --- |
| Age | 1.509 | < 0.001 |
| BMI | 0.984 | 0.049 |
| Immunodeficiency | 1.006 | 0.032 |
| SOFA d7 | 1.099 | <0.001 |
| Days Intubation to ECMO | 1.060 | <0.001 |
| Vt/PBW d7 | 0.998 | 0.015 |
| Respiratory frequency d7 | 0.994 | 0.636 |
| Driving pressure d7 | 1.014 | 0.417 |
| Inspiratory Pressure (P_high_) d7 | 1.057 | 0.004 |
| PEEP d7 | 1.048 | 0.025 |
| Compliance d7 | 0.990 | 0.033 |
| Dialysis d7 | 1.383 | 0.048 |
| pH d7 | 0.016 | <0.001 |
| Lactate d7 | 1.487 | <0.001 |
| ECMO Flow d7 | 1.464 | <0.001 |
| Sweep Gas Flow d7 | 1.182 | <0.001 |
| Tidal volume per predicted body weight d7 | 0.471 | 0.047 |

**Day 14**

| **Variable** | **Odds Ratio** | **p-value** |
| --- | --- | --- |
| Age | 1.509 | < 0.001 |
| BMI | 0.984 | 0.049 |
| Immunodeficiency | 1.006 | 0.032 |
| SOFA d14 | 1.121 | <0.001 |
| Days Intubation to ECMO | 1.060 | <0.001 |
| Vt/PBW d14 | 0.997 | <0.001 |
| Respiratory frequency d14 | 0.981 | 0.158 |
| Driving pressure d14 | 1.005 | 0.819 |
| Inspiratory Pressure (P_high_) d14 | 1.066 | 0.007 |
| PEEP d14 | 1.068 | 0.009 |
| Compliance d14 | 0.985 | 0.019 |
| Dialysis d14 | 1.808 | 0.001 |
| pH d14 | 0.011 | <0.001 |
| Lactate d14 | 1.952 | <0.001 |
| ECMO Flow d14 | 1.601 | <0.001 |
| Sweep Gas Flow d14 | 1.262 | <0.001 |
| Tidal volume per predicted body weight d14 | 0.742 | 0.002 |

All variables refer to the respective day of ECMO support.

Definition of Abbreviations: ECMO = extracorporeal membrane oxygenation; P_high_ = inspiratory pressure; PEEP = positive end expiratory pressure; Vt = tidal volume; PBW = predicted body weight; PaO_2_ = arterial partial pressure of oxygen; PaCO_2_ = arterial partial pressure of carbon dioxide, SOFA = sequential organ failure assessment.

**Table S2.** Multivariable Models all days

| **Model Variables** | **Odds Ratio (95%CI), p-value** | **Nagelkerke R^2^** |
| --- | --- | --- |
| **Day 1** | | |
| Age | 1.507 (1.332 – 1.705), < 0.001 | 0.082 |
| Lactate  on this day | 1.164 (1.072 – 1.265), < 0.001 |  |
| **Day 3** | | |
| Age | 1.544 (1.360 – 1.754), <0.001 | 0.096 |
| ECMO Flow  on this day | 1.398 (1.202 – 1.627), <0.001 |  |
| Lactate  on this day | 1.149 (1.012 – 1.304), 0.032 |  |
| **Day 5** | | |
| Age | 1.549 (1.256 – 1.770), <0.001 | 0.129 |
| ECMO Flow  on this day | 1.384 (1.185 – 1.173), <0.001 |  |
| Sweep Gas Flow  on this day | 1.092 (1.017 – 1.173), 0.016 |  |
| pH  on this day | 0.027 (0.003 – 0.288), 0.003 |  |
| **Day 7** | | |
| Age | 1.442 (1.214 – 1.714), <0.001 | 0.155 |
| SOFA-Score  on this day | 1.084 (1.037 – 1.132), <0.001 |  |
| Sweep Gas Flow  on this day | 1.205 (1.114 – 1.303), <0.001 |  |
| Lactate  on this day | 1.217 (1.010 – 1.467), 0.039 |  |
| **Day 14** | | |
| Tidal volume  on this day | 0.693 (0.564–0.851), < 0.001 | 0.185 |
| ECMO Blood Flow  on this day | 1.247 (1.004–1.548), 0.046 |  |
| Lactate  on this day | 2.442 (1.569–3.801),<0.001 |  |

Definition of Abbreviations: ECMO = extracorporeal membrane oxygenation; Vt = tidal volume; PBW = predicted body weight; PaO_2_ = arterial partial pressure of oxygen; SOFA = sequential organ failure assessment.

**Table S3.** Multivariable Day 14 Models including Respiratory Rate (as continuous and dummy variable with various thresholds) for all Patients still receiving ECMO support on day 14

| **Day 14 Model 3**  **(Forcing Respiratory Rate as continuous variable into the model)** | | | |
| --- | --- | --- | --- |
| **n=250** | | | |
|  | **Odds Ratio (95% CI)** | **p-value** | **Nagelkerke**  **R^2^** |
| Lactate  on this day | 2.447 (1.569-3.801) | <0.001 | 0.185 |
| ECMO blood flow  on this day | 1.247 (1.004-1.548) | 0.046 |  |
| Tidal volume  on this day | 0.814 (0.721-0.918) | <0.001 |  |
| Respiratory rate | 0.984 (0.956-1.013) | 0.284 |  |

| **Day 14 Model 4**  **(Forcing Respiratory Rate as dummy variable into the model)** | | | |
| --- | --- | --- | --- |
| **n=250** | | | |
|  | **Odds Ratio (95% CI)** | **p-value** | **Nagelkerke**  **R^2^** |
| Lactate  on this day | 2.472 (1.589-3.848) | <0.001 | 0.187 |
| ECMO blood flow  on this day | 1.233 (0.992-1.533) | 0.059 |  |
| Tidal volume  on this day | 0.818 (0.725-0.923) | 0.001 |  |
| Respiratory rate (≥12/min) | 0.549 (0.216-1.397) | 0.208 |  |

| **Day 14 Model 5**  **(Forcing Respiratory Rate as dummy variable into the model)** | | | |
| --- | --- | --- | --- |
| **n=250** | | | |
|  | **Odds Ratio (95% CI)** | **p-value** | **Nagelkerke**  **R^2^** |
| Lactate  on this day | 2.446 (1.559-3.773) | <0.001 | 0.195 |
| ECMO blood flow  on this day | 1.248 (1.005-1.551) | 0.045 |  |
| Tidal volume  on this day | 0.816 (0.723-0.921) | <0.001 |  |
| Respiratory rate (≥14/min) | 0.510 (0.258-1.011) | 0.054 |  |

| **Day 14 Model 6**  **(Forcing Respiratory Rate as dummy variable into the model)** | | | |
| --- | --- | --- | --- |
| **n=250** | | | |
|  | **Odds Ratio (95% CI)** | **p-value** | **Nagelkerke**  **R^2^** |
| Lactate  on this day | 2.398 (1.542-3.730) | <0.001 | 0.194 |
| ECMO blood flow  on this day | 1.256 (1.011-1.560) | 0.039 |  |
| Tidal volume  on this day | 0.814 (0.721-0.919) | <0.001 |  |
| Respiratory rate  (>16/min) | 0.581 (0.334-1.010) | 0.054 |  |

| **Day 14 Model 7**  **(Forcing Respiratory Rate as dummy variable into the model)** | | | |
| --- | --- | --- | --- |
| **n=250** | | | |
|  | **Odds Ratio (95% CI)** | **p-value** | **Nagelkerke**  **R^2^** |
| Lactate  on this day | 2.397 (1.540-3.730) | <0.001 | 0.192 |
| ECMO blood flow  on this day | 1.252 (1.008-1.555) | 0.042 |  |
| Tidal volume  on this day | 0.816 (0.723-0.921) | 0.001 |  |
| Respiratory rate  (>18/min) | 0.635 (0.382-1.056) | 0.080 |  |

| **Day 14 Model 8**  **(Forcing Respiratory Rate as dummy variable into the model)** | | | |
| --- | --- | --- | --- |
| **n=250** | | | |
|  | **Odds Ratio (95% CI)** | **p-value** | **Nagelkerke**  **R^2^** |
| Lactate  on this day | 2.442 (1.568-3.801) | <0.001 | 0.190 |
| ECMO blood flow  on this day | 1.245 (1.002-1.546) | 0.047 |  |
| Tidal volume  on this day | 0.809 (0.717-0.914) | 0.001 |  |
| Respiratory rate  (>20/min) | 0.668 (0.409-1.093) | 0.108 |  |

**Table S4.** Multivariable Day 14 Model including Respiratory Rate (≥ 14/min) and Respiratory System Compliance for all Patients still receiving ECMO support on day 14

| **Day 14 Model 9**  **(Forcing Respiratory Rate as dummy variable and respiratory system compliance into the model)** | | | |
| --- | --- | --- | --- |
| **n=250** | | | |
|  | **Odds Ratio (95% CI)** | **p-value** | **Nagelkerke**  **R^2^** |
| Lactate  on this day | 2.381 (1.528-3.709) | <0.001 | 0.191 |
| ECMO blood flow  on this day | 1.233 (0.990-1.535) | 0.061 |  |
| Tidal volume  on this day | 0.812 (0.694-0.950) | 0.009 |  |
| Respiratory rate  (>14/min) | 0.470 (0.223-0.950) | 0.035 |  |
| Compliance  on this day (mL/cmH_2_O) | 1.002 (0.981-1.022) | 0.877 |  |

**Figure S1.** Tidal Volumes and Driving Pressures during the ECMO runs


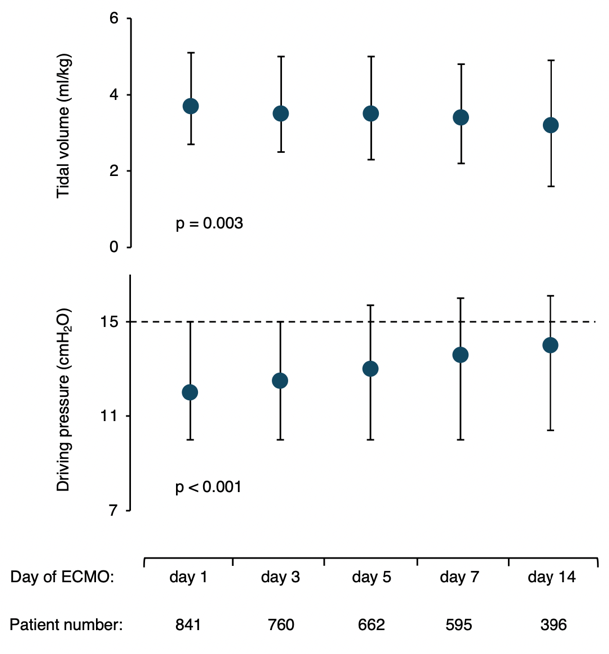


**Figure S1. Tidal Volumes and Driving Pressures during the ECMO runs**

Tidal volumes per predicted body weight and driving pressures for all patients under controlled ventilation receiving ECMO support on the respective day of their ECMO run.

Error bars represent interquartile ranges.

P-values were derived by repeated measures ANOVA analyses.
